# Real-Time Detection and Classification of Power Quality Disturbances

**DOI:** 10.3390/s22207958

**Published:** 2022-10-19

**Authors:** Mahsa Mozaffari, Keval Doshi, Yasin Yilmaz

**Affiliations:** Electrical Engineering Department, University of South Florida, Tampa, FL 33620, USA

**Keywords:** power quality disturbances, smart grid, anomaly detection, non-parametric sequential methods, sequential multi-hypothesis testing

## Abstract

This paper considers the problem of real-time detection and classification of power quality disturbances in power delivery systems. We propose a sequential and multivariate disturbance detection method (aiming for quick and accurate detection). Our proposed detector follows a non-parametric and supervised approach, i.e., it learns nominal and anomalous patterns from training data involving clean and disturbance signals. The multivariate nature of the method enables joint processing of data from multiple meters, facilitating quicker detection as a result of the cooperative analysis. We further extend our supervised sequential detection method to a multi-hypothesis setting, which aims to classify the disturbance events as quickly and accurately as possible in a real-time manner. The multi-hypothesis method requires a training dataset per hypothesis, i.e., per each disturbance type as well as the ’no disturbance’ case. The proposed classification method is demonstrated to quickly and accurately detect and classify power disturbances.

## 1. Introduction

Power quality (PQ) has become a major concern in power grids. The increasing penetration of renewable energy sources, increasing energy consumption, and the proliferation of modern electrical equipment are some of the sources of power quality disturbances (PQDs) that may cause major/minor damages to sensitive equipment and power system operations, such as blackouts. Due to the catastrophic damages caused by power losses to the safety, economy, and society, it is important to improve the grid’s reliability, security, and stability. To that end, the monitoring of the power system is crucial for assessing the PQ and overcoming the PQ problems in the system [1].

PQD, referring to the voltage/current quality, is the deviation of the voltage/current waveform from the ideal. In this paper, without loss of generality, we only consider the voltage quality disturbances. Voltage quality monitoring deals with analyzing the voltage waveform over time in order to detect and mitigate the voltage issues. Power quality monitoring allows for gaining better insights about the disturbances in the system, which in turn can help prevent potential damages, identify sources of disturbances, and make appropriate mitigating/preventive countermeasures in the system. Therefore, it is highly important to detect and identify the PQDs, as quickly and accurately as possible, so that the countermeasures could be taken in time. Fortunately, new technologies employed in a smart grid, such as high computational power and devices for real-time monitoring, communications, and automation, can facilitate the real-time detection and identification of the disturbances.

Although power quality monitoring has been studied for decades, new approaches are needed due to the emerging technological capabilities of smart grids and the integration of power grids with renewable energy resources and modern electrical equipment, such as electric vehicles and Internet-of-Things (IoT) devices.

## 2. Related Work

Many existing PQD detection and classification methods rely on frequency-domain or time-frequency domain analyses of the signals to extract informative features for further analysis to identify the type of disturbances, e.g., wavelet transform [2], Fourier transform, short-time Fourier transform (STFT), S-transform [3], etc. These methods are usually assisted with machine learning (ML)-based classification methods, including decision tree (DT) [3,4], support vector machine (SVM) [5,6], *k*-nearest-neighbor-based methods (*k*NN) [7,8,9], and neural networks [10,11,12,13,14,15,16,17,18].

In [16], the combination of S-transform-based feature extraction and a probabilistic neural network is used for the classification of eleven power quality disturbances. S-transform has an advantage over wavelet transform in detecting disturbances under noisy conditions. Reference [3] proposes extracting five features from the S-transform of the voltage waveform. These methods are effective in accurately classifying the disturbances; however, they lack the ability to be applied in real time due to their high computational complexity. While the problem of detection and classification of PQDs has been studied a lot, there is limited research on real-time approaches that focus on quick and accurate detection and classification. A real-time S-transform-based method has been proposed in [19], where the authors have proposed the use of dynamics to reduce the run-time of the transform and feature extraction. Despite the lower computational burdens of this method, it lacks the ability to quickly react to the disturbances due to the relatively large windows required by such methods. Although the proper window size is typically not discussed in the relevant literature, it is seen from the presented simulations that usually 10 or 12 cycles of the waveform are used to extract features in 50 and 60 Hz systems, respectively.

While the majority of existing works consider the concurrent detection and classification of PQDs, several other methods focus only on the detection, aimed at detecting the disturbances as quickly as possible. The methods in references [20] and [21] attempted to detect (as quickly and accurately as possible) after the PQD occurrences. These methods attempted to model the nominal and disturbance signals, employing techniques to deal with the unknown disturbance probability distributions. These methods are effective in detecting the PQDs very quickly and accurately; however, they do not provide any information regarding the type of the detected disturbances, and conventional classification methods are required to be further employed in order to help with the identification of PQDs. In this paper, we propose a method that is simple enough to be applied in real time and is able to quickly and accurately detect and classify the disturbances (we were motivated by the gap in accurate and timely joint detection and the classification of PQDs).

### Contributions

In summary, our contributions to this paper are as follows:

- The quick and accurate detection of PQDs in real-time; we propose a novel sequential, non-parametric, and supervised disturbance detector. The proposed detector, thanks to its multivariate nature, facilitates cooperative detection by multiple meters for coping with noisy measurements.
- The proposed detection method is proven to be asymptotically (as the training sets grow) optimal in the minimax sense in terms of minimizing the expected detection delay while satisfying a desired false alarm constraint.
- Extending the proposed detection method, a novel PQD detection and classification method is proposed, which is empirically shown to outperform the state-of-the-art techniques in terms of quickness and accuracy.

The remainder of the paper is organized as follows. Section 3 presents the system model for PQD detection and classification. Section 4.1 focuses on the derivation and analysis of the proposed sequential PQD detection method. Section 5.3 introduces the proposed joint detection and classification method for PQD. Finally, Section 6 concludes the paper with general remarks and future work directions.

## 3. System Model

Voltage waveform in the ideal form is a sinusoidal with constant frequency and magnitude, i.e.,

(1) s(t)=asin(2πft+ϕ),t∈R,

where *a*, *f*, and ϕ are the nominal magnitude, frequency, and phase angle, respectively. In practice, even in the nominal case without disturbance, the observed voltage values z(t)=s(t)+v(t) are distorted by the measurement noise v(t). After a disturbance occurs in the system, the voltage measurements become further distorted by an additional disturbance waveform δ(t), i.e., z(t)=s(t)+v(t)+δ(t). Therefore, we can view the voltage disturbance detection as the change in the distribution of the observed waveform. Let us define y(t) as the distortion signal added to the ideal waveform s(t). Before and after the occurrence of disturbance, y(t) consists of the noise v(t) and the noisy disturbance waveform measurements v(t)+δ(t), respectively. Since the ideal waveform parameters are deterministic and fixed, y(t) is easily calculated by subtracting the deterministic measurements s(t) from the voltage measurement z(t), i.e., y(t)=z(t)−s(t).

Assume that the voltage measurements are nominal initially, and an unknown disturbance occurs at an unknown time τ. The occurrence of a disturbance in the voltage waveform can be considered as a change in the distribution of the sampled observations:

(2) $$y_{n}=v_{n}∼P_{0},\;\;nS<\tau ;\;\;y_{n}=\delta_{n}+v_{n}∼P_{1},\;\;nS\geq \tau ,$$

where $y_{n}$ is the sampled observation at time n∈Z, *S* is the sampling period, $P_{0}$ is the probability distribution of pre-change observations, i.e., typically $N(0,\sigma^{2})$, $\delta_{n}$ is the disturbance at time *n*, and $P_{1}$ is the post-change probability distribution, which is unknown due to the fact that it depends on the type of disturbance occurring in the system. The objective of this problem is to detect a PQD as soon as possible and identify the type of PQD among a given list of known classes.

Sequential change detection (or change-point detection) methods are a class of statistical methods that have been extensively and successfully applied to many real-time applications (e.g., [21,22,23,24]) with the aim of detecting a change in the statistical distribution of the observations as quickly and accurately as possible after the occurrence of change in the observation [25]. In this paper, we aimed for the quick detection and classification of PQDs, and employed a sequential change detection approach for real-time detection and classification of PQDs.

## 4. Sequential Detection of Power Quality Disturbances

CUSUM is a well-known sequential change detection method that is applied in many application domains to detect changes in the statistical distribution of data [26]. CUSUM is optimal in the minimax sense [27] in terms of minimizing the detection delay (the time elapsed from the change time τ until the detection time *T*) while controlling the false alarm rate:

(3) $$\underset{T}{\inf}\underset{\tau }{\sup}\mathrm{ess}\underset{X_{\tau }}{\sup}E_{\tau }\left[{\left(T-\tau \right)}^{+}|X_{\tau }\right]\;\;s.t.\;\;E_{\infty }\left[H\right]\geq \beta .$$

In ((3)), $E_{\tau }$ represents the expectation given the change occurs at time τ, ${\left(.\right)}^{+}=\max\left(.,0\right)$, $E_{\infty }$ indicates the expectation given that the change never occurs, i.e., expected false alarm period. The  “ess sup” indicates essential supremum, which in practice is equivalent to supremum. To put it simply, the minimax performance criterion minimizes the average detection delay for the least favorable change-point τ and the least favorable history of measurements $X_{\tau }$ up to the change-point while the average false alarm period is constrained by β.

Despite being minimax optimal in minimizing the detection delay for a given false alarm constraint, CUSUM has the drawback of being parametric, i.e., it requires the perfect knowledge of the pre-change and post-change probability distributions and their parameters. Even if the correct probability distributions are known, the minimax optimality only holds asymptotically (as the available data size grows) when the parameters are estimated from data. The parametric nature of CUSUM limits its applicability in applications such as power quality monitoring in which the post-change parameters are typically unknown.

The non-parametric and data-driven methods on the other hand are suitable to deal with unknown probability distributions. A recent non-parametric and sequential anomaly detection method, called the online discrepancy test (ODIT), was proposed in [28]. It has been proven effective for achieving quick and accurate anomaly detection in real-world scenarios with many unknowns in the system model. However, ODIT is a semi-supervised method that only trains on nominal data. Even though this semi-supervised nature allows ODIT to be generic and not restricted to a certain list of anomaly types, it also prevents it from improving its performance on detecting known anomaly types by training on available data. Specifically, in PQD detection, a detector can be trained on sample data from the anomaly types of interest, as opposed to other real-world problems where obtaining anomalous training data are not tractable or desired. Hence, in this section, exploiting the sequential and data-driven properties of ODIT, we propose a novel supervised PQD detection method. In the next section, we further propose a multi-class extension for joint detection and classification.

### 4.1. Proposed Supervised Detection Method

Given the observed waveforms z(t) and y(t), the *d*-dimensional feature vector $x_{n}\in R^{d}$ is extracted using a time-domain or frequency-domain analysis during the time window [(n−1)S,nS]. Consider the nominal training set $X_{N}=\left\{x_{1},x_{2},\ldots ,x_{N}\right\}$ consisting of *N* nominal data points, as well as an anomaly training set $X_{M}^{\prime }=\left\{x_{1}^{\prime },x_{2}^{\prime },\ldots ,x_{M}^{\prime }\right\}$ containing *M* disturbance data points. Let us define $g_{i}\left(x_{n}\right)$ as the Euclidean distance between the observation $x_{n}$ and its *i*th nearest neighbor in $X_{N}$. Moreover, define $L_{n}$ as the sum of the *k* nearest neighbor (*k*NN) distances of observation $x_{n}$ with respect to the set $X_{N}$:

(4) $$L_{n}=\sum_{i=k-s+1}^{k}g_{i}\left(x_{n}\right),$$

where s∈{1,⋯,k} is a fixed number introduced for convenience. Similarly, $L_{n}^{\prime }$ denotes the total *k*NN distance of $x_{n}$ with respect to the anomaly train set $X_{M}^{\prime }$.

In the testing phase, our method computes the evidence for the anomaly in each observation $x_{n}$ by comparing the $L_{n}$ and $L_{n}^{\prime }$. This is in contrast with ODIT, which compares $L_{n}$ with a baseline statistic computed from nominal training data since it does not utilize any anomalous training data. Assuming sufficiently large nominal and anomaly sets, $x_{n}$ is more likely to be nominal if $L_{n}<L_{n}^{\prime }$, i.e., the observation is closer to the nominal dataset than the anomalous one. On the other hand, in the case of $L_{n}>L_{n}^{\prime }$, the observation is more likely to be anomalous. In the proposed supervised detector, the anomaly evidence for each observation is computed by:

(5) $$D_{n}=d\left(\log L_{n}-\log L_{n}^{\prime }\right)+\log\left(N/M\right),$$

where *d* is the dimensionality of data, and *N* and *M* are the sizes of the nominal and anomaly datasets, respectively. In practice, due to the inherent difficulty of acquiring anomalous observations, there is typically an imbalance between nominal and anomaly datasets. The *k*NN distances in a dense nominal dataset are expected to be smaller than those in a sparse anomaly dataset. Hence, log(N/M) serves as a correction factor, introduced to treat the imbalance between two datasets. In particular, log(N/M)>0 compensates for $L_{n}$ being unfairly smaller than $L_{n}^{\prime }$. $D_{n}$ denotes the positive/negative evidence for the anomaly. Negative $D_{n}$ suggests that the observation is more similar to the nominal dataset while the positive $D_{n}$ means the observation is more similar to the anomalous dataset. The update and stopping rules of the proposed method, given by

(6) $$\begin{array}{c} \begin{array}{cc} \Delta_{n} & =\max\left\{\Delta_{n-1}+D_{n},0\right\},\;\Delta_{0}=0,\\ T & =\min\{n:\Delta_{n}\geq h\}, \end{array} \end{array}$$

are similar to those of the ODIT and CUSUM. That is, it recursively updates a detection statistic $\Delta_{n}$ by accumulating the anomaly evidence over time and raising an alarm as soon as $\Delta_{n}$ exceeds a predefined threshold *h*, selected in a way to strike a balance between the detection delay and false alarm rates.

As the training datasets grow, the detector proposed in Equations (4)–(6) achieves asymptotic optimality in the minimax sense, as shown in the following theorem.

**Theorem** **1.** *When the nominal distribution $f_{0}\left(x_{n}\right)$ and anomalous distribution $f_{1}\left(x_{n}\right)$ are finite and continuous, as the training sets grow, the statistic $D_{n}$ given by (5) converges in probability to the log-likelihood ratio,*

(7) $$D_{n}\overset{p}{\to }\log\frac{f_{1}\left(x_{n}\right)}{f_{0}\left(x_{n}\right)}\;\;\;\mathrm{as}\;\;\;M,N\to \infty ,$$

*i.e., the method converges to CUSUM, which is minimax optimum in minimizing the expected detection delay while satisfying a false alarm constraint.*

**Proof.**  Consider a hypersphere $S_{t}\in R^{d}$ centered at $x_{n}$ with radius $g_{k}\left(x_{n}\right)$, the *k*NN distance of $x_{n}$ with respect to nominal set $X_{N}$. The maximum likelihood estimate for the probability of a point being inside $S_{t}$ under $f_{0}$ is given by k/N. It is known that, as the total number of points grows, this binomial probability estimate converges to the true probability mass in $S_{t}$ in the mean square sense [29], i.e., $k/N\overset{L^{2}}{\to }\int_{S_{t}}f_{0}\left(x\right)\;dx$ as N→∞. Hence, the probability density estimate ${\hat{f}}_{0}\left(x_{n}\right)=\frac{k/N}{V_{d}g_{k}{\left(x_{n}\right)}^{d}}$, where $V_{d}g_{k}{\left(x_{n}\right)}^{d}$ is the volume of $S_{t}$ with the appropriate constant $V_{d}$, converges to the actual probability density function, ${\hat{f}}_{0}\left(x_{n}\right)\overset{p}{\to }f_{0}\left(x_{n}\right)$ as N→∞ since $S_{t}$ shrinks and $g_{k}\left(x_{n}\right)\to 0$. Similarly, we can show that $\frac{k/M}{V_{d}g_{k}^{\prime }{\left(x_{n}\right)}^{d}}\overset{p}{\to }f_{1}\left(x_{n}\right)$ as M→∞, where $g_{k}^{\prime }\left(x_{n}\right)$ is the *k*NN distance of $x_{n}$ in the anomalous training set $X_{M}^{\prime }$. Hence, we conclude with $\log\frac{\frac{k/M}{V_{d}g_{k}^{\prime }{\left(x_{n}\right)}^{d}}}{\frac{k/N}{V_{d}g_{k}{\left(x_{n}\right)}^{d}}}=d\left[\log g_{k}\left(x_{n}\right)-\log g_{k}^{\prime }\left(x_{n}\right)\right]+\log\left(N/M\right)\overset{p}{\to }\log\frac{f_{1}\left(x_{n}\right)}{f_{0}\left(x_{n}\right)}\;\;\mathrm{as}\;\;M,N\to \infty$, where $L_{n}=g_{k}\left(x_{n}\right)$ and $L_{n}^{\prime }=g_{k}^{\prime }\left(x_{n}\right)$ for s=1.    □

**Remark** **1.** *In practice, the nominal and anomalous datasets may overlap. While the extent of overlap depends on the application, this may happen due to either the non-ideality of the feature space in terms of differentiating the nominal and anomalous data or the difficulty and inaccuracy inherent in anomalous data acquisition, e.g., some data points labeled as anomalous may be nominal in nature. For this reason, the proposed detector may require a pre-processing step, in which the anomalous dataset is cleaned of any data point, which is very similar to the nominal dataset. Specifically, given a statistical significance level α (e.g., 0.05), we eliminate any $x_{m}^{\prime }\in X_{M}^{\prime }$ from the anomalous training set whose total kNN distance is smaller than the ⌊Nα⌋th largest kNN distance in the nominal training set with respect to itself, i.e.,*

(8) $$X_{M}^{\mathrm{clean}}=X_{M}^{\prime }\backslash \left\{x_{m}^{\prime }\in X_{M}^{\prime }:L_{x_{m}^{\prime }}\leq L_{\left(N\alpha \right)}\right\},$$

*where ⌊·⌋ is the floor operator. Following the pre-processing step, in Equation (5), $L_{n}^{\prime }$ is calculated with respect to $X_{M}^{\mathrm{clean}}$, and M is the size of $X_{M}^{\mathrm{clean}}$.*

### 4.2. Simulation Results

**Figure 1:**
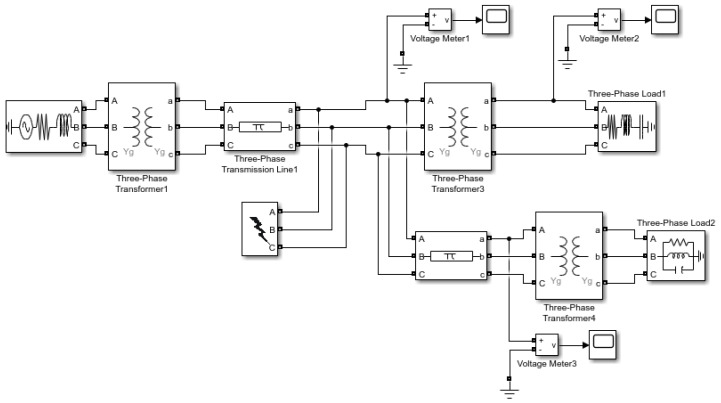
Simulink system for generating voltage sag disturbance induced by line fault.

**Figure 2:**
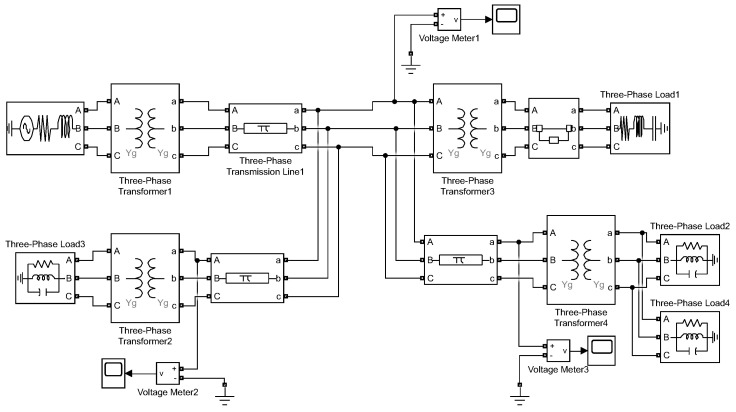
Simulink system for generating voltage swell disturbance induced by sudden load decrease.

**Figure 3:**
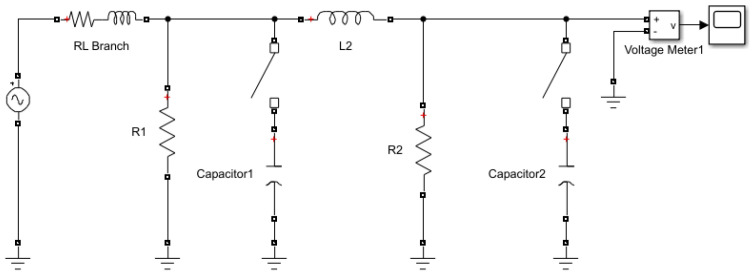
Simulink system for generating voltage oscillatory transient induced by capacitor switching.

In the simulations, we generate the disturbance signals using the Matlab/Simulink SimPowerSystems toolbox. Following [21], the voltage sag, swell, and oscillatory transient disturbances are induced by a distribution line fault, a sudden reduction in load, and capacitor bank switching, simulated by the circuits shown in Figure 1, Figure 2 and Figure 3). For example, in Figure 2, initially, the switch connecting Load 1 to the system is closed, and approximately at time 0.02 s the switch opens and the load of the system suddenly decreases. The voltage in the system is monitored through the three meters shown in the figure. In the experiments, the nominal waveform frequency is set to 60 Hz, normalized to the unit magnitude. The signal sampling frequency (at meters) is set to be 64 samples per cycle. The measurement noise variance is set to $\sigma^{2}=0.1$.

In this section, we apply the proposed detector to the detection of the common voltage disturbances: sag, swell, and oscillatory transients. We evaluate our proposed detector in terms of the average detection delay versus the false alarm rate and compare it with the semi-supervised ODIT [28] and the GLLR method proposed for sequential PQD detection in [21].

**Figure 4:**
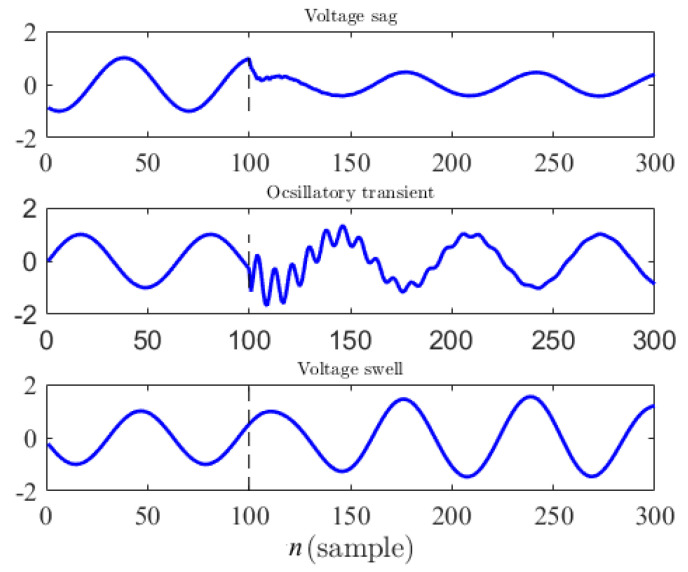
Voltage waveforms obtained from the circuits are shown in Figure 1, Figure 2 and Figure 3. Disturbances start at sample 101.

For evaluating the methods, we generated 2000 voltage waveforms for each disturbance type, where the disturbance occurs at sample 101 in the observations, e.g., Figure 4. After isolating the disturbance signal by subtracting the deterministic sine wave from the test waveform, we compute simple statistical features including average, standard deviation, RMS value, and auto-correlation within a moving window of size 5, shifted by 1 instance in time.

**Figure 5:**
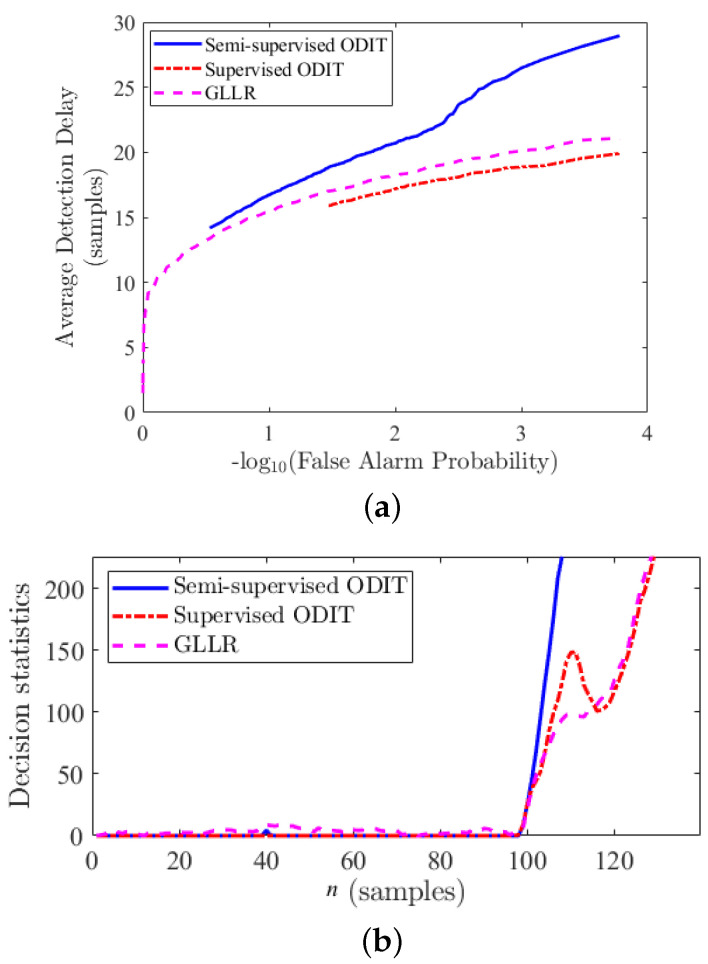
Comparison between the proposed supervised ODIT detector and competing methods GLLR [21] and semi-supervised ODIT [28]. (**a**) Performance comparison averaged over three voltage disturbance types, sag, swell, and oscillatory transients. (**b**) Sample decision statistic for the sag disturbance.

**Figure 6:**
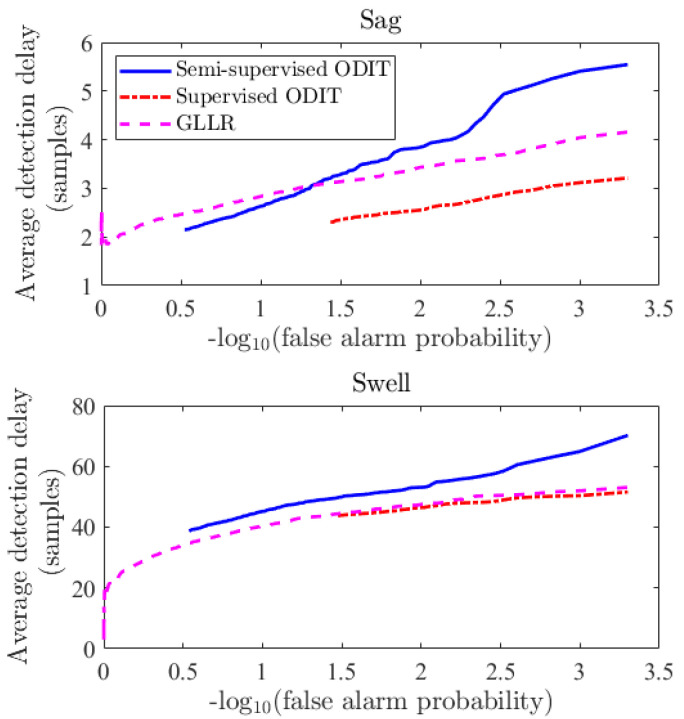

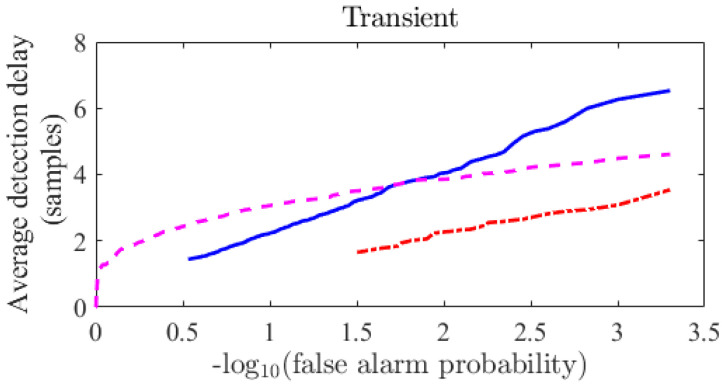
Performance comparison between the methods for detection of sag, transient, and swell disturbances with variance $\sigma^{2}=0.1$.

Figure 5a demonstrates the performance of the three methods, averaged over all three disturbance types, in terms of the average detection delay versus the probability of false alarm. We should note that all three methods detect the disturbances 100% of the time. The decision statistics of the methods (e.g., for voltage sag as depicted in Figure 5b) show an abrupt steady increase for all methods with the disturbance onset, whereas the average performance demonstrates that the proposed Supervised ODIT outperforms the GLLR and semi-supervised ODIT. Comparing the semi-supervised and supervised ODITs, we see that utilizing additional disturbance data improves the performance. Figure 6 depicts the average performance of the methods for each disturbance type individually. This figure confirms that supervised ODIT achieves the lowest detection delay for detecting all disturbance types. While all three detectors are able to detect the sag and transient disturbances in a few samples for practical false alarm rates, they need much more samples to detect the swell disturbance for the same level of false alarm rate. Due to this inherent difficulty in detecting the swell disturbance, the performance improvement of Supervised ODIT over the competing methods seems to be small on the linear scale. Its performance improvement is more clearly seen in the sag and transient cases. Since even very small thresholds for Supervised ODIT yield false alarm probabilities smaller than $10^{-1.5}$ (around 0.03) in these simulations, its delay performance for larger false alarm probabilities is not shown. Nevertheless, false alarm rates greater than 3% are usually not of interest in many applications.

**Figure 7:**
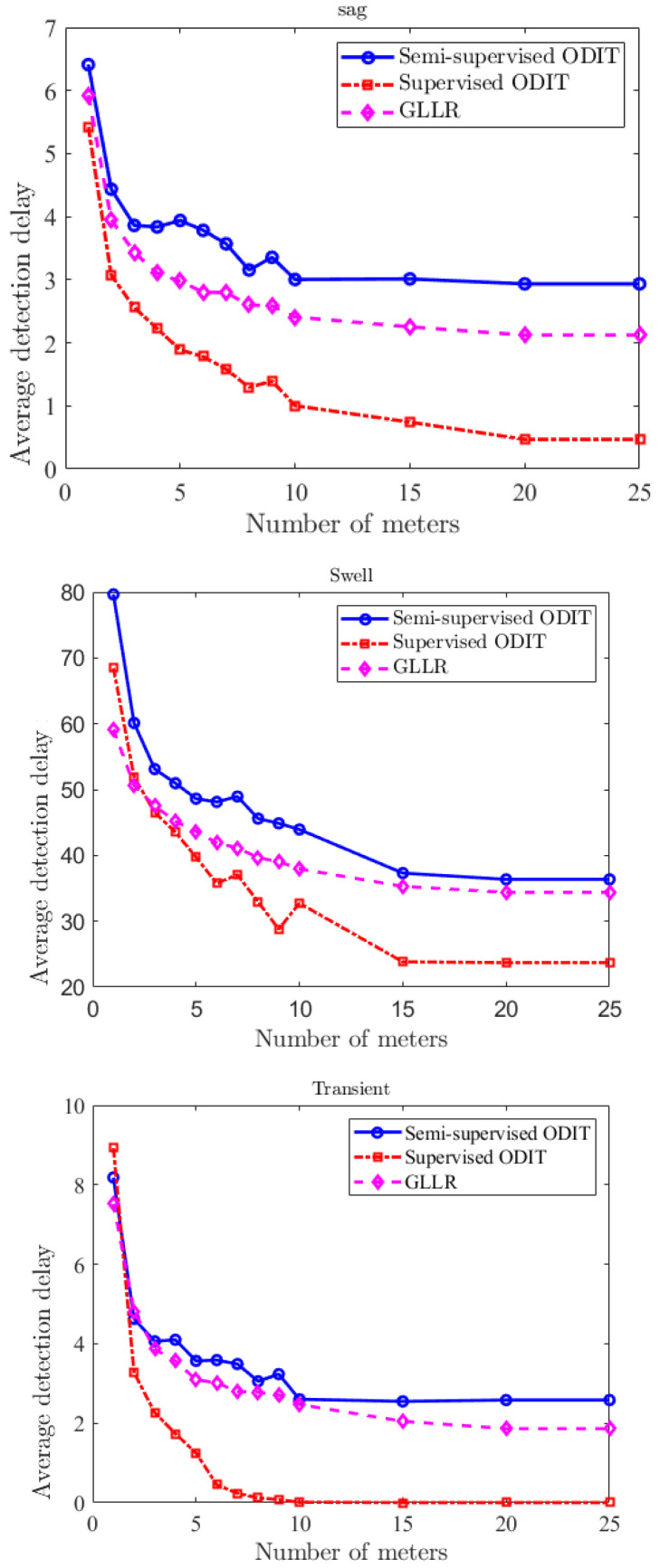
Average detection delay vs. number of meters for sag, sell, and transient disturbances. Detection delays are calculated for the fixed false alarm rate of 0.01.

Figure 7 demonstrates the performance improvement for the detection of sag, swell, and transient disturbances by the three methods as the number of meters employed in the system increases. It is seen that the performance of the proposed supervised ODIT detector improves faster and achieves a much smaller delay than the other two methods. The figures are obtained for a fixed false alarm rate of 0.01.

## 5. Classification of Power Quality Disturbances

Power quality disturbances, if not handled and mitigated properly, may cause serious damage to the grid. In order for proper and quick mitigation of the disturbance, it is important to identify the type of the event. Early identification of the event type would allow proper countermeasures to be taken in time. Thus, not only the accurate classification of the events are important, but also the quick classification of the disturbances is desirable. To that end, in this section, we consider the online classification of power quality disturbances as a sequential joint detection and classification problem, in which the goal is to detect a disturbance event in the observed system (and to accurately classify it as quickly as possible).

In the context of change detection, we can view online classification as a multi-hypothesis change detection problem, where there are several post-change hypotheses. Thus, the goal is to detect the change as quickly as possible and identify the post-change hypotheses correctly. Next, in Section 5.1, we formulate the problem of disturbance classification as a multi-hypotheses change detection problem, and in Section 5.2, Section 5.3 and Section 5.4 we present and evaluate our multi-hypothesis change detection method.

### 5.1. Problem Formulation

Consider a disturbance of type q∈Q happens at time τ and it changes the probability distribution *f* of the observed feature vector $x_{n}$. We formulate the problem as a multi-hypotheses change-detection problem, as:

(9) $$\begin{array}{cc} \; & f=f_{0},\;t<\tau ;\;f=f_{q}\left(\neq f_{0}\right),\;t\geq \tau ,q\in Q=\left\{1,\ldots ,Q\right\}, \end{array}$$

where *f* is the true probability distribution of the observations, $f_{0}$ is the nominal probability distribution, and $f_{q},q\in Q,$ is the post-change probability distribution for disturbance type *q*. The objective of this problem is to find the decision time *T* which minimizes the average detection delay while satisfying a constraint on the false alarm and false identification, which is equivalent to a classification error for the disturbance type:

(10) $$\begin{array}{c} \begin{array}{c} \underset{T}{\inf}\underset{q\in Q}{\sup}\underset{\tau }{\sup}\mathrm{ess}\underset{X_{\tau }}{\sup}E_{\tau }^{q}\left[{\left(T-\tau \right)}^{+}|X_{\tau }\right]\\ s.t.\;\;E_{\infty }^{q=0}\left[H\right]\geq \beta ,\;\;\underset{q\in Q}{\inf}\underset{\tau }{\inf}\underset{\hat{q}\in Q\backslash q}{\inf}E_{\tau }^{q}\left[\left(T^{\hat{q}}-\tau \right)\right]\geq \alpha , \end{array} \end{array}$$

where $E_{\tau }^{q}$ is the expectation given that change occurs at τ and post-change disturbance type is *q*, $E_{\infty }^{q=0}$ is the expectation given that no change occurs, and $T^{\hat{q}}$ is the time of false identification as type $\hat{q}\in Q\backslash q$. Put simply, this criterion aims to minimize the average detection delay for the least favorable change point, post-change hypothesis, and history of observations, while the average false alarm period is bounded by β, and the average worst-case false identification period is bounded by α.

### 5.2. Feature Extraction

Feature extraction is an important step toward the successful detection and classification of PQDs. It mainly aims to characterize the observed signal with lower dimensional data, i.e., extract useful information from sequential batches of the observed signal. For lightweight methods which can be deployed in real-time, it is important to compute simple features in rather small batches (i.e., time windows less than a cycle of sinusoidal signal). In this work, we employ statistical features that can be computed with small computational overhead while providing useful information to effectively distinguish between nominal and disturbance waveforms.

Given the observed voltage samples $z_{n}$ and isolated distortion samples $y_{n}=z_{n}-s_{n}$, where $s_{n}$ is the deterministic ideal waveform sample, the feature vector $x_{n}=\left[x_{n}^{1},\cdots ,x_{n}^{d}\right]$ is computed within a sliding window of size $w_{i}$ for each feature i=1,…,d. Specifically, at time instance *n*, the *i*th feature $x_{n}^{i}$ is computed using either $\left\{z_{n-w_{i}+1},\cdots ,z_{n}\right\}$ or $\left\{y_{n-w_{i}+1},\cdots ,y_{n}\right\}$. Note that unlike the existing methods in the literature, we calculate some features using the original voltage readings and the rest using the voltage distortion measurements. The features and their corresponding window sizes are given in Table 1. Features, such as the mean value, root mean square, standard deviation, autocorrelation, and entropy are commonly used statistical features used for PQD classification [30]. Waveform length is another time-domain feature mostly used in electromyographic (EMG) pattern recognition [31,32,33]. Zero crossing is a measure of the frequency of the signal in the time domain, which counts the number of times the voltage amplitude crosses zero. Waveform length measures the complexity of the signal within the window frame. We also introduce average fluctuation (AF), which measures the average of the absolute fluctuation value between consecutive points at which the slope of the signal changes. To calculate AF, as given in (11), first the set *I* of samples within the window frame at which the slope of signal changes is found. Next, AF is calculated as the average absolute change between consecutive indexes $m_{k}$$m_{k+1}$, where *k* refers to the index of elements in *I*, and $m_{k}$ denotes its time index.

### 5.3. Proposed Disturbance Classification Method: Vector-ODIT

A matrix-CUSUM method was proposed in [34] for online user activity detection. It performs multi-alternative change detection using a CUSUM-based method. Similar to CUSUM, matrix-CUSUM requires the probability distributions for all of the post-change disturbance types, which limits its applicability in PQD classification as the post-change disturbance parameters are typically unknown. Motivated by matrix-CUSUM, we here propose vector-ODIT based on the supervised ODIT detector introduced in Section 4.1. Vector-ODIT not only detects the onset of disturbance but also identifies the type of disturbance in a sequential and data-driven manner.

Assume Q={1,2,…,Q} is the set of post-change disturbance types, and we have Q+1 training datasets $\left\{X_{N_{q}}^{q},q\in 0\cup Q\right\}$, where $X_{N_{0}}^{0}$ is the nominal dataset of size $N_{0}$, and the rest are the datasets of size $N_{q}$ containing observations of disturbances of type q∈Q. For each *q*, we define the complement set $\tilde{q}=Q\backslash q$ and subsequently define the dataset $X_{N_{\tilde{q}}}^{\tilde{q}}=\cup_{j\in \tilde{q}}X_{N_{j}}^{j}$. For each observation at time *n*, the anomaly evidence $D_{n}^{q}$ for each q∈Q

(12) $$D_{n}^{q}=d\left(\log L_{n}^{\tilde{q}}-\log L_{n}^{q}\right)+\log\left(N_{\tilde{q}}/N_{q}\right),$$

where $L_{n}^{q}$ and $L_{n}^{\tilde{q}}$ are the total *k*NN distances of feature vector $x_{n}$ with respect to the datasets $X_{N_{q}}^{q}$ and $X_{N_{\tilde{q}}}^{\tilde{q}}$, respectively (see Equation (4)). According to Theorem 1, $D_{n}^{q}$ approximates the log-likelihood ratio $\log\frac{f_{q}\left(x_{n}\right)}{f_{\tilde{q}}\left(x_{n}\right)}$. Each element of the decision statistic vector $\Delta_{n}=\left[\Delta_{n}^{1},\cdots ,\Delta_{n}^{Q}\right]$ is recursively updated as

(13) $$\Delta_{n}^{q}=\max\left\{\Delta_{n-1}^{q}+D_{n}^{q},0\right\},\;\Delta_{0}^{q}=0.$$

(14) $$T=\min\{n:\Delta_{n}^{q}\geq h_{q},\;q=1,\cdots ,Q\},$$

and identifies the disturbance type as the index *q* which causes the alarm. The vector-ODIT algorithm is summarized in Algorithm 1.
**Algorithm 1** The proposed vector-ODIT procedure for PQD classification1:*Input:*$k,s,\alpha ,\left\{X_{N_{0}}^{0},\cdots ,X_{N_{Q}}^{Q}\right\},\left\{h_{1},\cdots ,h_{Q}\right\}$2:*Initialize:*$\Delta \leftarrow 0^{Q\times 1},\;n\leftarrow 0$3:*Training phase:*4:Clean datasets according to (8).5:*Test phase:*6:**while**$\Delta_{n}^{q}<h_{q},\forall q\in Q$**do**7:     n←n+18:    Obtain new voltage observation $z_{n}$, compute distortion value $y_{n}$, and compute features $x_{n}$ of Table 1.9:     For each q∈Q, compute $D_{n}^{q}$ and $\Delta_{n}^{q}$ as in Equations (12) and (13).10:Declare PQD at time *n* and identify the type as *q* for which $\Delta_{n}^{q}\geq h_{q}$.

### 5.4. Simulation Results

In this section, we evaluate our PQD classification method in terms of classifying the disturbances into four classes, voltage sag, swell, oscillatory transient, and harmonics, using MATLAB. Following the common practice in the literature, signals are generated synthetically using the following equation [35]

(15) $$z\left(t\right)=\delta_{1}\left(t\right)\sin\left(2\pi ft\right)+\delta_{2}\left(t\right).$$

For voltage sag and swell, $\delta_{1}\left(t\right)\neq 0$ and $\delta_{2}\left(t\right)=0$. Specifically,

$$\delta_{1}\left(t\right)=1-a\left[u\left(t-t_{1}\right)-u\left(t-t_{2}\right)\right]$$

for sag, and

$$\delta_{1}\left(t\right)=1+a\left[u\left(t-t_{1}\right)-u\left(t-t_{2}\right)\right]$$

for swell, where u(t) denotes the unit step function, a∈[0.1,0.8] is randomly selected from uniform distribution, and the starting and ending times are also randomly chosen as $t_{2}-t_{1}\in \left[T,9T\right]$ (T=1/f). For each PQD class, as well as the nominal class ($\delta_{1}\left(t\right)=\delta_{2}\left(t\right)=0$), we generate signals of length 10 cycles with fundamental frequency of f=50 Hz (i.e., T=0.02 s) and sampling frequency of 50×64 Hz. For transient and harmonics disturbances, $\delta_{1}\left(t\right)=0$ and $\delta_{2}\left(t\right)\neq 0$. Specifically,

$$\delta_{2}\left(t\right)=\sum_{i\in \{3,5,7\}}k_{i}\sin\left(i2\pi ft\right)$$

for harmonics, and

$$\delta_{2}\left(t\right)=ae^{-(t-t_{1})/\tau }\left[u\left(t-t_{1}\right)-u\left(t-t_{2}\right)\right]\sin\left(j2\pi f\left(t-t_{1}\right)\right)$$

for transient, where all parameters are uniformly random with $k_{i}\in \left[0.05,0.3\right]$, τ∈ [3 ms, 50 ms], j∈{6,⋯,18}, a∈[0.3,0.5], and $t_{2}-t_{1}\in \left[0.5T,3T\right]$. We populate the per class training datasets by performing feature extraction according to Section 5.2 within moving window blocks of the specified sizes, shifted by 1 point at a time. The proposed classification method does not need any training process, but the training datasets are needed to be cleaned according to (8) in order to remove the overlapping data instances.

**Figure 8:**
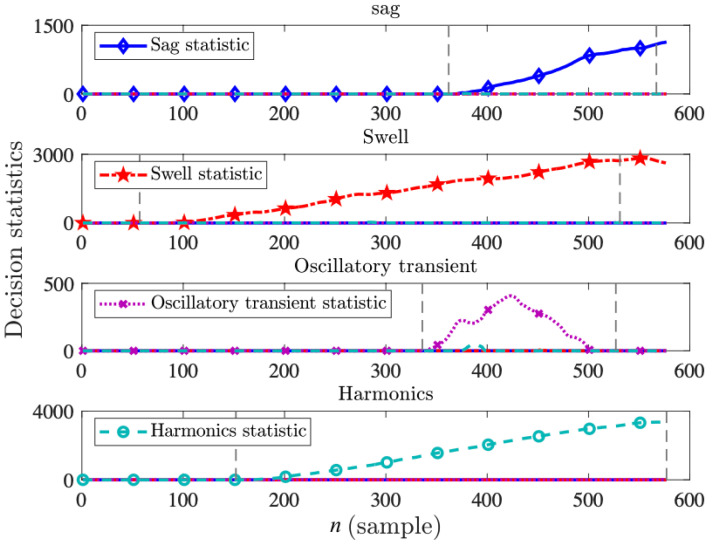
Decision statistics of vector-ODIT for four voltage disturbance types: sag, swell, oscillatory transient, and harmonics. The disturbance onset and ending times are shown with vertical dashed gray lines. When the disturbance starts, the corresponding decision statistic successfully increases steadily, while the other decision statistics remain around zero.

During the test phase, 200 signals of duration 0.2 s per each disturbance type are generated randomly, i.e., the signal parameters, such as the disturbance starting and ending time, magnitude, and phase are selected uniformly random within the allowed range. Figure 8 shows four sample paths for the decision statistics vector $\Delta_{n}$ over time. The onset and end of each disturbance in the signal are shown with the vertical gray dashed lines in the figures. As the figures suggest, after the occurrence of the disturbance, the decision statistic corresponding to the correct disturbance type starts to increase persistently, leading to the detection and classification by the corresponding threshold. Whereas, the other three decision statistics (representing the cumulative evidence for the other disturbance types) remain zero or fluctuate subtly above zero. The selection of proper thresholds is of crucial importance to strike the desired balance between the false alarm rate, classification accuracy, and classification delay. We empirically set the thresholds (given in Equation (14)) to maximize the classification accuracy while also keeping the delay suitable for real-time decision-making. Typically, smaller thresholds would result in smaller detection/classification delays, but also larger false alarm rates and lower classification accuracy, and vice versa for larger thresholds. In simulations, setting the four thresholds to proper values, we achieve 0.0038 false alarm rate and 98.38% classification accuracy with the detection/classification delay of 39.46 data samples on average, as shown in Table 2. The misclassifications are mainly due to the failure to detect the oscillatory transient disturbance signals or misclassifying them as harmonics. Note that by vector-ODIT, the detection and classification happen at the same time. The additional classification capability comes with some degree of larger delays compared to the detection-only results reported in Section 4.2.

In Table 3, the average performance of the proposed method in terms of the classification accuracy for each disturbance type is compared with several state-of-the-art methods in the literature. The accuracy of each method has been reported for noisy conditions with signal-to-noise ratio (SNR) value being 20 (or higher as reported in the corresponding paper). To evaluate the real-time detection and classification capability of methods, we also present the average delay performance in terms of waveform cycle in Table 3. The proposed method achieves the presented accuracy in less than one cycle for each disturbance type. The overall average delay of 39.46 samples, shown in Table 2, corresponds to 0.61 cycles. However, the existing methods in the literature except [30] require multiple waveform cycles, typically 10–12, to extract features from frequency-domain analysis such as Fourier, wavelet, and S transform. Furthermore, in the existing works, how to run the proposed methods sequentially is not discussed. Hence, we consider moving their feature extraction windows by the window length after analyzing and classifying each batch. This makes these methods considerably (around 10 times) slower than the proposed method in terms of detecting and classifying PQDs. To calculate the exact average delay values for these methods, we need to know how many disturbance samples are required in the feature extraction window for successful detection and classification. Since such information is not reported in [6,14,16,19,35,36,37,38,39], we assume that at least one cycle of the disturbance is required to be in the feature extraction window. Therefore, we approximate the average delay as 5.5 cycles, 10 cycles in the worst case, and 1 cycle in the best case.

The FFT and ANN methods [30], as opposed to the other existing methods, uses 16 time-domain and frequency-domain features computed in windows of size 1 cycle (or 128 samples) shifted by one time unit at each time. Although it achieves above 90% classification accuracy, we should note that it considers relatively low noise levels with SNR changing between 35 and 40 dB). Our proposed vector-ODIT method, on the other hand, achieves above 98% classification accuracy for a higher noise level of 20 dB. We also tested vector-ODIT under 30 dB. With this lower noise level, it is able to achieve 100% accuracy and a 0% false alarm rate. Moreover, feature extraction proposed in FFT & ANN [30] relies on the calculation of total harmonic distortion of the signals, up to the 25th harmonic, which is much more computationally expensive than the features Vector-ODIT uses.

## 6. Conclusions

Detecting and classifying power quality disturbances (PQD) in a timely and accurate manner was considered. A novel data-driven sequential detector was proposed and its asymptotic optimality in terms of minimizing the average detection delay in the minimax sense was proven. Through voltage disturbance simulations, we showed that the proposed method outperforms the existing sequential detectors, ODIT and GLLR, in terms of quick detection while satisfying the same false alarm rate. We also proposed a novel sequential classifier by extending the proposed detector to the multi-hypothesis testing setup. The performance of the proposed classifier was evaluated on four voltage disturbance types (sag, swell, oscillatory transient, and harmonics) by comparing it with a number of existing methods. For all disturbance types, it achieved accurate classification (98.38% accuracy with 0.38% false alarm rate under 20 dB SNR, and 100% accuracy with 0% false alarm under 30 dB SNR) within a period of less than a waveform cycle (on average 0.61 cycle, which corresponds to 39.46 samples or 0.0123 s). Thanks to its sequential design, it is much quicker than the existing methods, which typically take more than 5 cycles to achieve the same accuracy levels.

## Figures and Tables

**Table 1:** Description of the extracted features. Features defined over $y_{n}$ values use the isolated distortion observations while others defined over $z_{n}$ values use the original voltage meter readings. 𝟙{·} denotes the indicator function, which takes the value 1 when the inner argument is true and 0 otherwise. In (11), *k* is the index for the set I; $m_{k}$ is the time index of the *k*th element in I; and |I| denotes the number of elements in I.

	Feature	Equation	Window Size
1	Distortion at time *n*	$y_{n}$	$w_{1}=1$
2	Root mean square (RMS)	$RMS=\sqrt{\frac{1}{w_{2}}\sum_{m=0}^{w_{2}-1}y_{n-m}^{2}}$	$w_{2}=64$
3	Standard deviation	$\sigma =\sqrt{\frac{\sum_{m=0}^{w_{3}-1}{\left(y_{n-m}-\bar{y}\right)}^{2}}{w_{3}-1}}$	$w_{3}=64$
4	Autocorrelation	$R=\frac{\sum_{m=1}^{w_{4}}\left(z_{n-m}-\bar{z}\right)\left(z_{n-m+1}-\bar{z}\right)}{\sum_{m=0}^{w_{4}-1}{\left(z_{n-m}-\bar{z}\right)}^{2}}$	$w_{4}=64$
5	Entropy	$E=\sum_{m=0}^{w_{5}-1}\log y_{n-m}^{2}$	$w_{5}=64$
6	Waveform length	$WL=\sum_{m=1}^{w_{6}}\left|z_{n-m+1}-z_{n-m}\right|$	$w_{6}=64$
7	Zero crossing	$ZC=\sum_{m=1}^{w_{7}}𝟙\left\{\left(z_{n-m}\times z_{n-m+1}\right)<0\right\}$	$w_{7}=64$
8	Average fluctuation	(11) $$\begin{array}{c} cAF=\frac{1}{\left|I\right|}\sum_{k=1}^{\left|I\right|}\left|y_{m_{k}+1}-y_{m_{k}}\right|,\\ I=\{m:1\leq m\leq w_{8},\\ \left(y_{n-m+1}-y_{n-m}\right)\left(y_{n-m}-y_{n-m-1}\right)<0\} \end{array}$$	$w_{8}=64$

**Table 2:** The performance of the vector-ODIT in terms of classification delay. The thresholds are set in a way to achieve the maximum classification accuracy and the minimum false alarm probability.

Disturbance Type	Classification Delay in Samples (and in Seconds)
Sag	26.68 (0.0083 s)
Swell	34.62 (0.0108 s)
Oscillatory transient	45.59 (0.0142 s)
Harmonics	51.23 (0.0160 s)
Overall average	39.46 (0.0123 s)

**Table 3:** Performance comparison for classification of sag, swell, oscillatory transient, and harmonics. The performances are presented in terms of the classification accuracy, the average delay of correct classification, and false alarm (false classification of the normal signal as a disturbance).

	Classification Accuracy/Average Delay (Cycle)						
**Classification Method**	**Sag**	**Swell**	**Transient**	**Harmonics**	**Average**	**Normal**	**SNR (dB)**
ADALINE & FFNN [15]	98/5.5	99/5.5	86/5.5	90/5.5	94.3/5.5	-	20
ST & PNN [16]	98/5.5	92/5.5	86/5.5	95/5.5	92/5.5	100	20
FFT & ANN [30]	91.64/0.84	96.46/0.84	92.37/0.84	97.74/0.84	93.49/0.84	-	35
DRST & DAG-SVM [35]	99/5.5	98.5/5.5	97.5/5.5	99.5/5.5	98.33/5.5	100	20
Dynamics & ST [19]	95/5.5	97/5.5	97/5.5	97/5.5	96.33/5.5	96	20
TQWT & MSVM [6]	98/5.5	100/5.5	94/5.5	100/5.5	97.33/5.5	-	20–50
WT & SVM [36]	89/5.5	89/5.5	98/5.5	97/5.5	92/5.5	-	20
SSD Hybrid Dict. [37]	100/5.5	100/5.5	100/5.5	100/5.5	100/5.5	100	30
Deep CNN [38]	99.20/5	100/5	99.50/5	100/5.5	99.56/5.5	97.70	20
VMD & DT [39]	98.2/5.5	97.6/5.5	98.2/5.5	98.5/5.5	98/5.5	100	30
**Proposed**	99/0.41	100/0.54	95.5/0.71	99/0.80	98.38/0.61	99.62	20
	100/0.41	100/0.54	100/0.71	100/0.80	100/0.61	100	30

## Data Availability

Not applicable.
